# Association of Vitamin D Status and Other Clinical Characteristics With COVID-19 Test Results

**DOI:** 10.1001/jamanetworkopen.2020.19722

**Published:** 2020-09-03

**Authors:** David O. Meltzer, Thomas J. Best, Hui Zhang, Tamara Vokes, Vineet Arora, Julian Solway

**Affiliations:** 1 Department of Medicine, University of Chicago, Chicago, Illinois; 2 Center for Health and the Social Sciences, University of Chicago, Chicago, Illinois

## Abstract

**Importance:**

Vitamin D treatment has been found to decrease the incidence of viral respiratory tract infection, especially in patients with vitamin D deficiency. Whether vitamin D is associated with coronavirus disease 2019 (COVID-19) incidence is unknown.

**Objective:**

To examine whether the last vitamin D status before COVID-19 testing is associated with COVID-19 test results.

**Design, Setting, and Participants:**

This retrospective cohort study at an urban academic medical center included patients with a 25-hydroxycholecalciferol or 1,25-dihydroxycholecalciferol level measured within 1 year before being tested for COVID-19 from March 3 to April 10, 2020.

**Exposures:**

Vitamin D deficiency was defined by the last measurement of 25-hydroxycholecalciferol less than 20 ng/mL or 1,25-dihydroxycholecalciferol less than 18 pg/mL before COVID-19 testing. Treatment changes were defined by changes in vitamin D type and dose between the date of the last vitamin D level measurement and the date of COVID-19 testing. Vitamin D deficiency and treatment changes were combined to categorize the most recent vitamin D status before COVID-19 testing as likely deficient (last level deficient and treatment not increased), likely sufficient (last level not deficient and treatment not decreased), and 2 groups with uncertain deficiency (last level deficient and treatment increased, and last level not deficient and treatment decreased).

**Main Outcomes and Measures:**

The outcome was a positive COVID-19 polymerase chain reaction test result. Multivariable analysis tested whether vitamin D status before COVID-19 testing was associated with testing positive for COVID-19, controlling for demographic and comorbidity indicators.

**Results:**

A total of 489 patients (mean [SD] age, 49.2 [18.4] years; 366 [75%] women; and 331 [68%] race other than White) had a vitamin D level measured in the year before COVID-19 testing. Vitamin D status before COVID-19 testing was categorized as likely deficient for 124 participants (25%), likely sufficient for 287 (59%), and uncertain for 78 (16%). Overall, 71 participants (15%) tested positive for COVID-19. In multivariate analysis, testing positive for COVID-19 was associated with increasing age up to age 50 years (relative risk, 1.06; 95% CI, 1.01-1.09; *P* = .02); non-White race (relative risk, 2.54; 95% CI, 1.26-5.12; *P* = .009), and likely deficient vitamin D status (relative risk, 1.77; 95% CI, 1.12-2.81; *P* = .02) compared with likely sufficient vitamin D status. Predicted COVID-19 rates in the deficient group were 21.6% (95% CI, 14.0%-29.2%) vs 12.2%(95% CI, 8.9%-15.4%) in the sufficient group.

**Conclusions and Relevance:**

In this single-center, retrospective cohort study, likely deficient vitamin D status was associated with increased COVID-19 risk, a finding that suggests that randomized trials may be needed to determine whether vitamin D affects COVID-19 risk.

## Key Points

**Question:**

Is vitamin D status, reflecting vitamin D levels and treatment, associated with test results for coronavirus disease 2019 (COVID-19)?

**Findings:**

In this cohort study of 489 patients who had a vitamin D level measured in the year before COVID-19 testing, the relative risk of testing positive for COVID-19 was 1.77 times greater for patients with likely deficient vitamin D status compared with patients with likely sufficient vitamin D status, a difference that was statistically significant.

**Meaning:**

These findings appear to support a role of vitamin D status in COVID-19 risk; randomized clinical trials are needed to determine whether broad population interventions and interventions among groups at increased risk of vitamin D deficiency and COVID-19 could reduce COVID-19 incidence.

## Teaser

This cohort study examines whether patients’ most recent vitamin D levels and treatment for insufficient vitamin D levels are associated with test results for coronavirus disease 2019 (COVID-19).

## Introduction

Coronavirus disease 2019 (COVID-19), caused by the severe acute respiratory syndrome coronavirus 2 (SARS-CoV-2), often produces severe lower respiratory symptoms and has caused more than 745 000 deaths worldwide.^1^ One challenge in halting this pandemic is the absence of evidence demonstrating effective pharmacologic interventions to prevent COVID-19. Vitamin D treatment has been identified as a potential strategy to prevent or treat COVID-19.^2^ Vitamin D treatment has been found to decrease other viral respiratory infections, especially in persons with vitamin D deficiency.^3^ Vitamin D deficiency is common, affecting nearly half the US population, with higher rates among persons with darker skin or reduced sun exposure, including persons living in higher latitudes in the winter, nursing home residents, and health care workers.^4^ COVID-19 is more prevalent among African American individuals,^5^ persons living in northern cities in the late winter,^6^ older adults,^7^ nursing home residents,^8^ and health care workers,^9^ populations who all have increased risk of vitamin D deficiency.^4,10,11,12^ Moreover, COVID-19 is less prevalent in pregnant women and children^13^ and in persons living in Japan,^14^ in whom rates of vitamin D deficiency are lower.^15,16,17^ Shelter-in-place orders to reduce the spread of COVID-19 may also decrease sun exposure, potentially increasing needs for vitamin D supplementation.^18^ Given the low risks and low cost of vitamin D treatment, recent reporting has suggested that vitamin D treatment should be scaled.^19,20^ Nevertheless, evidence of whether vitamin D deficiency is associated with COVID-19 infection and whether vitamin D treatment may help decrease the burden and spread of COVID-19 is lacking.

Using data from the electronic health record at the University of Chicago Medicine (UCM) in Chicago, Illinois, we hypothesized that persons tested for COVID-19 would be more likely to test positive for COVID-19 if they had likely deficient vitamin D levels than if they had likely sufficient vitamin D levels. Because patients may have experienced changes in their vitamin D treatment after their most recent vitamin D level measurement before COVID-19 testing, we combined data on patients’ last vitamin D level before COVID-19 testing and changes in their treatment after that vitamin D level measurement to construct a measure of vitamin D status indicating whether each patient was expected to have a vitamin D level that was deficient, sufficient, or of uncertain sufficiency at the time they were tested for COVID-19.

## Methods

### Participants

We obtained data for all 4314 patients tested for COVID-19 at UCM from March 3 to April 10, 2020. We obtained electronic health record data for demographic, comorbidity, laboratory, and medication data within 1 year before the date of their first COVID-19 test. Vitamin D levels and treatments within 14 days of COVID-19 testing were excluded from analyses to avoid possible confounding by potential early manifestations of COVID-19, eg, presenting for health care with symptoms that could lead to testing for and treatment of vitamin D deficiency. Twenty-two patients were excluded from eligibility from the analytic sample because their only vitamin D level measurement occurred within 14 days of COVID-19 testing. This study was approved by the University of Chicago Biological Sciences Division Institutional Review Board with a waiver of consent for use of identifiable data. It was determined that this analysis could not be reliably executed without the use of identifiable data and that it was impracticable to obtain consent from all subjects. This study followed the Strengthening the Reporting of Observational Studies in Epidemiology (STROBE) reporting guideline for cohort studies.

### Measurements

All variables were defined based on information from the UCM electronic health record (Epic; Epic Systems). COVID-19 test status was determined by any positive COVID-19 polymerase chain reaction test result, with the Centers for Disease Control and Prevention^21^ or Viacor^22^ test used until in-house testing with the test from Roche (cobas) began on March 15, 2020.^23^ Because of test supply, testing at UCM was limited to persons presenting with potential symptoms of COVID-19 admitted to the hospital or health care workers with COVID-19 symptoms and exposure. Patients were deemed to be vitamin D deficient if their most recent serum vitamin D levels within 1 year before their first COVID-19 tests were less than 20 ng/mL for 25-hydroxycholecalciferol (to convert to nanomoles per liter, multiply by 2.496) or less than 18 pg/mL for 1,25-dihydroxycholecalciferol (to convert to picomoles per liter, multiply by 2.4) and deemed not deficient if their most recent levels were equal to or greater than 20 ng/mL or equal to or greater than 18 pg/mL, respectively.^24,25,26,27^ Vitamin D treatment was defined by report in the electronic health record of vitamin D either in the patient medication list or prescription orders. Vitamin D3 dosing was defined based on most recent daily dose recorded over the past year excluding the 14 days before testing: none, 1 to 1000 IU or a multivitamin, 2000 IU, or greater than or equal to 3000 IU. Indicators for treatment with vitamin D2 and calcitriol were also included. We accounted for possible changes in patients’ vitamin D treatment after the time of their last vitamin D level by categorizing changes in treatment between the date of the last vitamin D level and 14 days before COVID-19 testing as increased, unchanged, or decreased according to the following ordering: calcitriol was considered the highest treatment category followed in decreasing order by greater than or equal to 3000 IU D3, 2000 IU D3, D2, 1-1000 IU D3 or multivitamin, and no vitamin D. We then combined the data on last vitamin D level measurements with changes in treatment after that last vitamin D level to assign each patient to 1 of 4 categories reflecting their likelihood of being vitamin D deficient at the time of COVID-19 testing: likely deficient (last level deficient and treatment not increased), likely sufficient (last level not deficient and treatment not decreased), and 2 groups with uncertain deficiency (last level deficient and treatment increased, and last level not deficient and treatment decreased).

Age, sex, and race/ethnicity were also obtained from the electronic health record and coded as reported in Table 1. We also obtained the most recent data during the study period up to 14 days before COVID-19 testing to calculate body mass index and the following *International Statistical Classification of Diseases, Tenth Revision, Clinical Modification (ICD-10-CM)–*based Elixhauser comorbidity clusters^28^ potentially related to COVID-19 and/or vitamin D metabolism: hypertension, diabetes, chronic pulmonary disease, pulmonary circulation disorders, depression, immunosuppression, liver disease, and chronic kidney disease (eAppendix in the Supplement). *ICD-10-CM* codes were drawn over a 2-year period because of evidence that a 2-year lookback improves diagnosis capture compared with a 1-year lookback.^29^

**Table 1.** Characteristics of Patient Population

Characteristic	No. (%)			*P* value^a^
	Full sample	Vitamin D deficient		
		Yes (<20 ng/mL)	No (≥20 ng/mL)	
No. of patients	489	172	317	
Age, y				
Mean (SD)	49.2 (18.4)	45.9 (17.6)	51.0 (18.6)	.004^b^
<50	260 (53)	109 (63)	151 (48)	.004
50-64	122 (25)	33 (19)	89 (28)	
≥65	107 (22)	30 (17)	77 (24)	
Sex				
Female	366 (75)	133 (77)	233 (74)	.38
Male	123 (25)	39 (23)	84 (27)	
Race				
White	158 (32)	30 (17)	128 (40)	<.001
Other than White	331 (68)	142 (83)	189 (60)	
Ethnicity				
Hispanic	41 (8)	14 (8)	27 (9)	>.99
Non-Hispanic	448 (92)	158 (92)	290 (91)	
Employee status, UCM employee				
Yes	161 (33)	59 (34)	102 (32)	.69
No	328 (67)	113 (66)	215 (68)	
Vitamin D level evaluated in past year	489 (100)	172 (100)	317 (100)	
Most recent vitamin D <20 ng/mL	172 (35)	172 (100)	0	
Interpretation				
Likely deficient^c^	124 (25)	124 (72)		
Uncertain deficiency^d^	48 (10)	48 (28)		
Uncertain deficiency^e^	30 (5)		30 (9)	
Likely sufficient^f^	287 (59)		287 (91)	
Days since most recent vitamin D level				
Mean	162	156	166	.30
Median	151	129	159	.10
Comorbidity indicators				
Hypertension	261 (53)	89 (52)	172 (54)	.64
Diabetes	137 (28)	51 (30)	86 (27)	.60
Chronic pulmonary disease	117 (24)	43 (25)	74 (23)	.74
Pulmonary circulation disorders	20 (4)	9 (5)	11 (3)	.35
Depression	119 (24)	45 (26)	74 (23)	.51
Chronic kidney disease	116 (24)	36 (21)	80 (25)	.32
Liver disease	56 (11)	17 (10)	39 (12)	.46
Comorbidities with immunosuppression	105 (21)	36 (21)	69 (22)	.91
BMI				
Mean	29.8	30.4	29.4	.22^b^
≥30	229 (47)	88 (51)	141 (44)	.18
Most recent active vitamin D treatment before COVID-19 test				<.001
None	212 (43)	80 (47)	132 (42)	.34
1-1000 IU D3/multivitamin	113 (23)	28 (16)	85 (27)	.01
2000 IU D3	60 (12)	7 (4)	53 (17)	<.001
≥3000 IU D3	20 (4)	10 (6)	10 (3)	.16
D2	76 (16)	44 (26)	32 (10)	<.001
Calcitriol	8 (2)	<5^g^	5 (2)	>.99
Test positive for COVID-19	71 (15)	32 (19)	39 (12)	.06

Abbreviations: BMI, body mass index (calculated as weight in kilograms divided by height in meters squared); COVID-19, coronavirus disease 2019; UCM, University of Chicago Medicine.

^a^ P values were determined using the Fisher exact test except where otherwise indicated.

^b^ *t* Test.

^c^ Answer was yes to most recent vitamin D level within 1 year being deficient (<20 ng/mL); dose was stable or decreased after last visit. Vitamin D dose was rank-ordered as follows: calcitriol > 3000+ IU D3 > 2000 IU D3 > D2 > 1-1000 IU D3/multivitamin > no vitamin D.

^d^ Answer was yes to most recent vitamin D level within 1 year being deficient (<20 ng/mL); dose was increased after last visit. Vitamin D dose was rank-ordered as follows: calcitriol > 3000+ IU D3 > 2000 IU D3 > D2 > 1-1000 IU D3/multivitamin > no vitamin D.

^e^ Answer was no to most recent vitamin D level within 1 year being deficient (<20 ng/mL); dose was decreased after last visit. Vitamin D dose was rank-ordered as follows: calcitriol > 3000+ IU D3 > 2000 IU D3 > D2 > 1-1000 IU D3/multivitamin > no vitamin D.

^f^ Answer was no to most recent vitamin D level within 1 year being deficient (<20 ng/mL); dose was stable or increased after last visit. Vitamin D dose was rank-ordered as follows: calcitriol > 3000+ IU D3 > 2000 IU D3 > D2 > 1-1000 IU D3/multivitamin > no vitamin D.

^g^ Frequency counts of 5 or less have been masked in this table to preserve confidentiality.

### Statistical Analysis

Basic descriptive statistics were reviewed for all variables. In comparing patients with last vitamin D levels that were deficient and patients with last levels that were not deficient, Fisher exact test was used for binary variables and the *t* test for continuous variables. A multivariable generalized linear model with binomial residuals and log-link function^30^ was estimated with the covariates noted above. Statistical significance was defined as *P* < .05. All tests were 2-tailed.

## Results

### Patient Characteristics

Of 4314 patients tested for COVID-19 during the study period, 499 had a vitamin D level measured in the year before testing, and 489 had complete data and were included in our analytic sample, with mean (SD) age of 49.2 (18.4) years, 366 (75%) female participants, and 331 (68%) of race other than White (Table 1). Of the 331 persons with recorded race other than White, race was Black or African American for 286 (86%) and Asian/Mideast Indian for 24 (7%), and 21 (6%) reported multiple race. One hundred and seventy-two (35%) had vitamin D deficiency. The Figure depicts the distribution of the most recent vitamin D level measured between 1 year before and 14 days before COVID-19 testing by COVID-19 test result and shows smaller numbers of patients distributed across the categories with nondeficient vitamin D levels compared with the deficient category.

**Figure.**
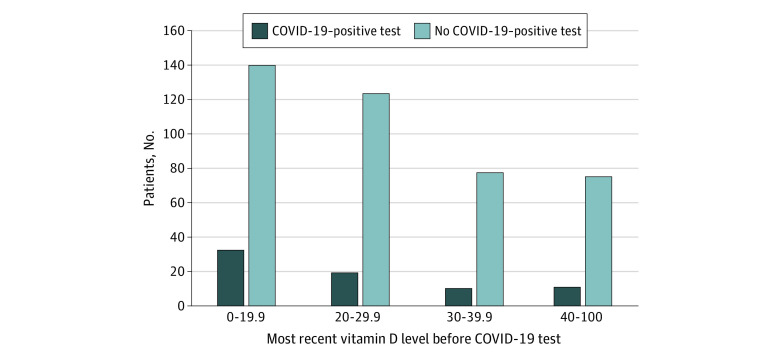
Most Recent Vitamin D Levels Before COVID-19 Test

Table 1 presents descriptive statistics for comorbidity measures, vitamin D treatments, and rates of testing positive for COVID-19 and stratifies all of the statistics reported by whether the last vitamin D level was deficient. Compared with patients who were not vitamin D deficient, patients who were vitamin D deficient were more likely to be younger (age 45.9 years vs 51.0 years; *P* = .004), race other than White (142 of 172 [83%] vs 189 of 317 [60%]; *P* < .001), and receive vitamin D2 (44 of 172 [26%] vs 32 of 317 [10%]; *P* < .001) and less likely to receive vitamin D3 (45 of 172 [26%] vs 148 of 317 [47%]; *P* < .001).

Combining vitamin D deficiency and treatment after the most recent vitamin D level to assess vitamin D status before COVID-19 testing, 124 (25%) patients were likely deficient, 287 (59%) were likely sufficient, and 48 (10%) and 30 (6%) were in the 2 groups with uncertain deficiency. eTable 1 in the Supplement presents the same descriptive statistics as Table 1, stratified by the 4 vitamin D status categories used in our multivariable analysis. The findings are similar except that there are statistically significant differences across the 4 categories in terms of employment status, median days since most recent vitamin D level, and the proportion with diabetes. Omitting the groups with uncertain vitamin D status from our analysis did not change our findings.

Among the 124 patients who had a deficient last vitamin D level and did not have vitamin D treatment increased, 113 (91%) continued to receive no vitamin D or their prior dose of vitamin D (Table 2). Among the 48 patients with a deficient last vitamin D level who had an increase in treatment after that level, 38 (79%) transitioned from no vitamin D treatment to vitamin D2 or vitamin D3, 20 (42%) transitioned from no vitamin D treatment to vitamin D2 and 18 (38%) transitioned from no vitamin D treatment to vitamin D3 or multivitamin, while 6 (12%) patients changed from vitamin D2 or a low dose of vitamin D3 to a higher dose of vitamin D3 (Table 3).

**Table 2.** Vitamin D Treatment Changes Among the Patients Who Had a Deficient Last Vitamin D Level and Did Not Have Vitamin D Treatment Increased (N = 124)

Treatment after vitamin D level	None	1-1000 IU D3/multivitamin	D2	2000 IU D3/calcitriol
Treatment before vitamin D level				
None	78	0	0	0
1-1000 IU D3/multivitamin	0	7	0	0
D2	<5^a^	<5^a^	21	0
2000 IU D3/calcitriol	0	<5^a^	5	7

^a^ Frequency counts of 5 or less have been masked in this table to preserve confidentiality.

**Table 3.** Vitamin D Treatment Changes Among the Patients Who Had a Deficient Last Vitamin D Level and an Increase in Treatment After That Level (N = 48)

Treatment after vitamin D level	None	1-1000 IU D3/multivitamin	D2	2000 IU D3/Calcitriol
Treatment before vitamin D level				
None	0	8	20	10
1-1000 IU D3/multivitamin	0	0	<5^a^	<5^a^
D2	0	0	0	<5^a^
2000 IU D3/calcitriol	0	0	0	0

^a^ Frequency counts of 5 or less have been masked in this table to preserve confidentiality.

### Follow-up and Outcomes

Overall, 71 (15%) participants tested positive for COVID-19. Among the 172 (35%) participants whose most recent vitamin D level was deficient, 32 (19%) tested positive for COVID-19 compared with 39 (12%) for participants whose last vitamin D level was not deficient (*P* = .06).

Table 4 shows the results of the multivariable generalized linear model for testing positive for COVID-19.^31,32^ Patients with likely deficient vitamin D status at the time of COVID-19 testing had an increased relative risk of testing positive for COVID-19 (relative risk, 1.77; 95% CI, 1.12-2.81; *P* = .02) compared with patients with likely sufficient status at the time of COVID-19 testing, for an estimated mean rate in the deficient group of 21.6% (95% CI, 14.0%-29.2%) vs 12.2% (95% CI, 8.9%-15.4%) in the sufficient group. Testing positive for COVID-19 was also associated with increasing age up to age 50 years (relative risk, 1.06; 95% CI, 1.01-1.09; *P* = .02), and with race other than White (relative risk, 2.54; 95% CI, 1.26-5.12; *P* = .009) and was not associated with comorbidities except for a decreased risk in persons with conditions associated with immunosuppression (relative risk, 0.39; 95% CI, 0.20-0.76; *P* = .005). Estimated risk of testing positive for COVID-19 was not significantly different for either patient group classified as having uncertain vitamin D status compared with patients with either likely deficient or likely sufficient status, but point estimates for the 2 uncertain status groups were between those of likely deficient and likely sufficient groups and had wide confidence intervals.

**Table 4.** Multivariable Association of Vitamin D Deficiency and Treatment With Testing Positive for COVID-19 in 489 Patients

Characteristic	No. (%)	Relative risk (95% CI)	*P* value
Age (linear spline)^a^			
<50	260 (53)	1.05 (1.01-1.09)	.02
≥50	229 (47)	1.02 (1.00-1.05)	.06
Sex			
Male	123 (25)	1 [Reference]	
Female	366 (75)	0.87 (0.52-1.44)	.58
Race			
White	158 (32)	1 [Reference]	
Other than White	331 (68)	2.54 (1.26-5.12)	.009
Ethnicity			
Non-Hispanic	448 (92)	1 [Reference]	
Hispanic	41 (8)	0.29 (0.04-2.01)	.21
Employee status, UCM employee			
No	328 (67)	1 [Reference]	
Yes	161 (33)	0.93 (0.52-1.64)	.79
Most recent vitamin D <20 ng/mL			
Likely deficient^b^	124 (25)	1.77 (1.12-2.81)	.02
Uncertain deficiency^c^	48 (10)	1.10 (0.49-2.43)	.82
Uncertain deficiency^d^	30 (5)	1.09 (0.43-2.82)	.85
Likely sufficient^e^	287 (59)	1 [Reference]	
Comorbidity indicators			
Hypertension	261 (53)	1.08 (0.60-1.97)	.79
Diabetes	137 (28)	0.78 (0.49-1.26)	.31
Chronic pulmonary disease	117 (24)	0.91 (0.55-1.52)	.73
Pulmonary circulation disorders	20 (4)	0.64 (0.23-1.79)	.40
Depression	119 (24)	1.22 (0.74-2.02)	.44
Chronic kidney disease	116 (24)	0.80 (0.49-1.32)	.39
Liver disease	56 (11)	0.99 (0.47-2.08)	.98
Comorbidities with immunosuppression	105 (21)	0.39 (0.20-0.76)	.005
BMI, mean	29.8	1.02 (0.996-1.048)	.10
Goodness-of-link test of squared predicted value^31^	NA	NA	.87
Hosmer-Lemeshow goodness-of-fit decile test^32^	NA	NA	.89

Abbreviations: BMI, body mass index (calculated as weight in kilograms divided by height in meters squared); COVID-19, coronavirus disease 2019; NA, not applicable; UCM, University of Chicago Medicine.

^a^ A piecewise linear spline with a single knot at 50 improved model fit over models with unadjusted age or more complex parameterizations.

^b^ Answer was yes to most recent vitamin D level within 1 year being deficient (<20 ng/mL); dose was stable or decreased after last visit. Vitamin D dose was rank-ordered as follows: calcitriol > 3000+ IU D3 > 2000 IU D3 > D2 > 1-1000 IU D3/multivitamin > no vitamin D.

^c^ Answer was yes to most recent vitamin D level within 1 year being deficient (<20 ng/mL); dose was increased after last visit. Vitamin D dose was rank-ordered as follows: calcitriol > 3000+ IU D3 > 2000 IU D3 > D2 > 1-1000 IU D3/multivitamin > no vitamin D.

^d^ Answer was no to most recent vitamin D level within 1 year being deficient (<20 ng/mL); dose was decreased after last visit. Vitamin D dose was rank-ordered as follows: calcitriol > 3000+ IU D3 > 2000 IU D3 > D2 > 1-1000 IU D3/multivitamin > no vitamin D.

^e^ Answer was no to most recent vitamin D level within 1 year being deficient (<20 ng/mL); dose was stable or increased after last visit. Vitamin D dose was rank-ordered as follows: calcitriol > 3000+ IU D3 > 2000 IU D3 > D2 > 1-1000 IU D3/multivitamin > no vitamin D.

### Sensitivity Analysis

Our results were robust to controlling for the number of days from last vitamin D level to COVID-19 testing. They also were consistent when the analysis was performed separately for non-White and White persons, although the small number of White persons forced the removal of some covariates for model estimation in that subgroup, specifically ethnicity, chronic pulmonary disease, pulmonary circulation disorders, chronic kidney disease, liver disease, and an indicator of uncertain vitamin D deficiency status. Because hypertension, obesity, and diabetes may be responsive to vitamin D treatment, sensitivity analyses were also performed omitting these factors as covariates, which did not significantly change these findings.

## Discussion

To our knowledge, this study provides the first assessment of the association of vitamin D deficiency and potentially insufficient treatment with testing positive for COVID-19. The multivariable analysis suggests that persons who are likely to have deficient vitamin D levels at the time of COVID-19 testing were at substantially higher risk of testing positive for COVID-19 than were persons who were likely to have sufficient levels. That patients with deficient last vitamin D levels who did have increased treatment were not found to have increased risk for COVID-19 compared with patients with likely sufficient vitamin D status may suggest a protective effect of treatment, but the confidence intervals on estimated rates for these groups are too wide to exclude the possibility of no treatment effect.

Our findings about the increased risk of testing positive for COVID-19 with likely deficient vitamin D status compared with likely sufficient vitamin D status contrasts with the findings of a recent study by Hastie et al.^33^ That article examined the association between vitamin D deficiency and testing positive for COVID-19 within the UK Biobank and did not find a statistically significant association. However, the vitamin D levels studied were between 10 and 14 years before the COVID-19 diagnosis, and the analysis did not control for treatment after the levels were assessed. When we examined our data by limiting vitamin D levels to those that were more distant or did not account for treatment, we also found weaker associations of deficient vitamin D levels with testing positive for COVID-19. The findings of Hastie et al^33^ may therefore reflect limitations of the data and analytic approach they applied.

Our results raise the consideration of whether treatment for vitamin D deficiency is associated with reductions in the risk of COVID-19. Since vitamin D deficiency may be increased by many factors that could be associated with COVID-19 risk, including age, obesity, diabetes, and chronic illness more generally, observed associations of vitamin D with outcomes in almost any observational study may fail to accurately reflect any potential causal effects of vitamin D on outcomes. Nevertheless, our analysis controls for many of these factors, and the idea that adequate vitamin D levels could prevent COVID-19 is supported by the meta-analysis of randomized clinical trials by Martineau et al^3^ that found vitamin D treatment of persons with vitamin D deficiency can reduce other viral respiratory infections, among which coronaviruses are common causative organisms. Although that meta-analysis suggests benefits of vitamin D supplementation in people who are vitamin D deficient, it also reports smaller but statistically significant effects of supplementation even in people whose vitamin D levels are sufficient by current standards. This finding is important because those standards are largely based on needs of bone health since needs for immune function are not known. These findings suggest that randomized clinical trials with varying doses of vitamin D may be warranted in populations with and without vitamin D deficiency to understand if vitamin D reduces the risk of COVID-19.

The low costs of vitamin D and its general safety, at least at doses of up to 4000 IU per day,^34^ support arguments for population-level supplementation, perhaps for targeting groups at high risk for vitamin D deficiency and/or COVID-19, as noted above. Since African American and Hispanic populations in the US have both high rates of vitamin D deficiency and bear a disproportionate burden of morbidity and mortality from COVID-19,^35,36^ they may be particularly important populations to engage in studies of whether vitamin D can reduce the incidence and burden of COVID-19. Testing of vitamin D levels may be an important tool in guiding treatments, and the availability of low-cost home testing for vitamin D may be valuable given the benefits of social distancing in COVID-19.

If vitamin D does reduce COVID-19 incidence, it is tempting to consider whether it might reduce COVID-19 transmission. Vitamin D strengthens innate immunity, so it might be expected to decrease COVID-19 infection and transmission.^37^ Vitamin D also affects metabolism of zinc,^38^ which decreases replication of coronaviruses.^39^ However, caution is required because of the potential importance of asymptomatic persons in COVID-19 spread. Vitamin D modulates immune function through effects on dendritic cells and T cells,^40^ which may promote viral clearance and reduce inflammatory responses that produce symptoms. Higher vitamin D levels correlate with lower interleukin 6 levels, which are a major target for controlling cytokine storm in COVID-19.^41,42^ To the extent that it prevents infection, decreases viral replication, or accelerates viral clearance, vitamin D treatment might reduce spread. On the other hand, if vitamin D reduces inflammation, it might increase asymptomatic carriage and decrease symptomatic presentations, including cough, making it hard to predict its effect on viral spread.

### Limitations

This study has limitations. First, vitamin D deficiency may be a consequence associated with a range of chronic health conditions or behavioral factors that plausibly increase COVID-19 risk. Nevertheless, the results are robust to including a broad set of demographic and comorbidity indicators that have either physiological reasons for consideration or have been suggested to influence COVID-19 outcomes. Moreover, neither patients who were deficient in vitamin D and had increased treatment nor patients who were not deficient in vitamin D who had decreased treatment were more likely than patients who were not vitamin D deficient and at least maintained their current treatment (ie, had nondeficient status) to test positive for COVID-19. If the observed association were due to confounding by behavioral or other health factors, such associations might have been expected, although our limited sample size might be inadequate to identify such effects. An additional limitation is that our data are limited to those available in the UCM electronic health record. Patterns of vitamin D screening, treatment, or COVID-19 testing at UCM or in other institutions might have somehow selected for patients who induced an association between observed vitamin D status and testing positive for COVID-19. We considered whether specific versions of this broad range of alternative hypotheses might explain our findings, including the idea that vitamin D treatment not recorded at UCM prior to COVID-19 testing might have biased our results. Analysis of medication information reported at the time of COVID-19 testing did not identify changes in vitamin D dosing. Another limitation is that only a few individuals received higher doses of vitamin D3 or had relative high vitamin D levels, limiting power to assess whether vitamin D dose or levels are associated with the likelihood of COVID-19 (Table 1, Figure; eTable 2 in the Supplement). We also included calcitriol levels in defining vitamin D deficiency and included patients treated with vitamin D2 or calcitriol, which are often used in patients with chronic kidney disease or hypoparathyroidism. In sensitivity analysis, our results were robust to omitting these patients. We also note that our sample is enriched in persons with vitamin D deficiency because of the large number of African American individuals, adults with chronic illness, and health care workers, all living in a northern city and exposed to COVID-19 during winter. Vitamin D deficiency is highly prevalent in the US but could be a smaller risk factor in other populations. The relative simplicity of the analysis performed here would facilitate replication of this analysis in other settings.

## Conclusions

The findings of this study suggest a role of vitamin D status, based on deficiency of levels and treatment, in risk of COVID-19 infection. Randomized clinical trials of interventions to reduce vitamin D deficiency are needed to determine if those interventions could reduce COVID-19 incidence, including both broad population interventions and interventions among groups at increased risk of vitamin D deficiency and/or COVID-19.

## Notes

Supplement. **eAppendix.** Methods **eTable 1.** Characteristics of Patient Population **eTable 2.** Bivariate Analysis of Most Recent Active Vitamin D Treatment 14 Days Before COVID-19 Test Order and COVID-19 Test Results, Among Patients With Most Recent Vitamin D in Past Year <20 ng/mL **eReference**
