# Supplementary material for: Association of Vitamin D Status and Other Clinical Characteristics With COVID-19 Test Results
Source: JAMA Netw Open. 2020 Sep 3;3(9):e2019722. doi: 10.1001/jamanetworkopen.2020.19722 (PMC7489852; doi:10.1001/jamanetworkopen.2020.19722)
Supplement: Supplement. — eAppendix. Methods eTable 1. Characteristics of Patient Population eTable 2. Bivariate Analysis of Most Recent Active Vitamin D Treatment 14 Days Before COVID-19 Test Order and COVID-19 Test Results, Among Patients With Most Recent Vitamin D in Past Year <20 ng/mL eReference [file jamanetwopen-e2019722-s001.pdf]

Supplementary Online Content

Meltzer DO, Best TJ, Zhang H, Vokes T, Arora V, Solway J. Association of vitamin D status and other clinical characteristics with COVID-19 test results. *JAMA Netw Open*. 2020;3(9):e2019722. doi:10.1001/jamanetworkopen.2020.19722

eAppendix. Methods
eTable 1. Characteristics of Patient Population
eTable 2. Bivariate Analysis of Most Recent Active Vitamin D Treatment 14 Days Before COVID-19 Test Order and COVID-19 Test Results, Among Patients With Most Recent Vitamin D in Past Year <20 ng/mL
eReference

This supplementary material has been provided by the authors to give readers additional information about their work.

© 2020 Meltzer DO et al. *JAMA Network Open.*

eAppendix. Methods

Chronic condition indicators. Each chronic condition indicator was set equal to 1 if the patient had at least one of a list of ICD-10-CM diagnosis codes included on an administrative/billing record with a discharge date between January 1, 2018 and April 10, 2020. The lists of codes used for the indicators was defined using the HCUP Elixhauser Comorbidity Software.[1] For each condition, we used all ICD-10-CM and DRG codes listed within categories in the Elixhauser file comformat_icd10_cm_2020_1.sas, entitled Creation of Format Library for Comorbidity Groups, ICD-10-CM Elixhauser Comorbidity Software, Version 2020.1. The actual ICD-10-CM codes can be found by finding the following category variable names in the file. Hypertension: HTN, HHRWCHF, HHRWHRF, HHRWOHRF, HHRWRF, HRENWORF, HRENWRF, HTNCX, HTNPREG, HTNWCHF, HTNWOCHF, OHTNPREG, HTNCXDRG, HTNDRG. Diabetes: DM, DMCX, DIABDRG. Chronic pulmonary disease: CHRNLUNG, PULMDRG. Pulmonary circulation disorders: PULMCIRC. Depression: DEPRESS. CKD: RENLFAIL, HHRWCHF, HHRWHRF, HHRWOHRF, HHRWRF, HRENWRF, RENALDRG, RENFDRG. Liver disease: LIVER. CM_IS: AIDS, METS, TUMOR, ARTH.

© 2020 Meltzer DO et al. *JAMA Network Open.*

eTable 1: Characteristics of Patient Population												
	Full sample			Likely deficient[a]		Uncertain deficiency: increased treatment[b]		Uncertain deficiency: decreased treatment[c]		Likely sufficient[d]		Fisher's exact test for difference
	N	%		N	%	N	%	N	%	N	%	p-value
Number of patients	489	100		124	100	48	100	30	100	287	100	
Age												
years, mean (SD)	49.2 (18.4)			46.0 (18.1)		45.8 (16.4)		50.2 (20.0)		51.1 (18.5)		0.04 (ANOVA)
<50	260	53		77	62	32	67	10	33	141	49	
50-64	122	25		25	20	8	17	14	47	75	26	0.01
65+	107	22		22	18	8	17	6	20	71	25	
Gender												0.27
Female	366	75		93	75	40	83	19	63	214	75	
Male	123	25		31	25	8	17	11	37	73	25	
Race												<0.001
White	158	32		22	18	8	17	8	27	120	42	<0.001
Other than White	331	68		102	82	40	83	22	73	167	58	
Ethnicity												0.53
Hispanic	41	8		8	6	6	13	<5		24	8	
Not Hispanic	448	92		116	94	42	88	27	90	263	92	
Employee status												0.007
UChicago employee	161	33		41	33	18	38	<5		100	35	
Not UChicago employee	328	67		83	67	30	63	28	93	187	65	
Vitamin D level evaluated in past year	489	100		124	100	48	100	30	100	287	100	
Most recent vitamin D<20 ng/ml	172	35		124	100	48	100	0	0	0	0	
Days since most recent vitamin D level (mean)	162			149		174		134		169		0.09 (ANOVA)
Days since most recent vitamin D level (median)	151			117		165		112		161		0.04
Comorbidity indicators												
Hypertension	261	53		65	52	24	50	21	70	151	53	0.30
Diabetes	137	28		30	24	21	44	11	37	75	26	0.04
Chronic pulmonary disease	117	24		34	27	9	18	8	27	66	23	0.62
Pulmonary circulation disorders	20	4		6	5	<5		<5		10	3	0.67
Depression	119	24		37	30	8	17	11	37	63	22	0.08
Chronic kidney disease	116	24		27	22	9	18	12	40	68	24	0.17
Liver disease	56	11		13	10	<5		8	27	31	11	0.09
Comorbidities with immunosuppression	105	21		28	23	8	17	7	23	62	22	0.85
BMI (mean)	29.8			30.0		31.5		28.8		29.5		0.40 (ANOVA)
BMI $\geq$30 kg/m^2	229	47		58	47	30	63	12	40	129	45	0.13
Most recent active vitamin D treatment before COVID-19 test												<0.001
None	212	43		70	56	10	21	8	27	124	43	<0.001
1-1000 IU D3/Multivitamin	113	23		18	15	10	21	15	50	70	24	0.001
2000 IU D3	60	12		<5		<5		<5		52	18	<0.001
3000+ IU D3	20	4		<5		7	15	<5		8	3	0.004
D2	76	16		27	22	17	35	<5		28	10	<0.001
Calcitriol	8	2		<5		0	0	0	0	5	2	0.85
Test positive COVID-19	71	15		26	21	6	13	<5		35	12	0.15

Frequency counts of less than 5 have been replaced with the notation <5 to reduce the potential to identify members of the study sample.

[a]Answer was yes to most recent vitamin D level within 1 year being deficient (<20 ng/mL); dose was stable or decreased after last visit. Vitamin D dose was rank-ordered as follows: calcitriol > 3000+ IU D3 > 2000 IU D3 > D2 > 1-1000 IU D3/multivitamin > no vitamin D.

© 2020 Meltzer DO et al. *JAMA Network Open.*

[b]Answer was yes to most recent vitamin D level within 1 year being deficient (<20 ng/mL); dose was increased after last visit. Vitamin D dose was rank-ordered as follows: calcitriol > 3000+ IU D3 > 2000 IU D3 > D2 > 1-1000 IU D3/multivitamin > no vitamin D.
[c]Answer was no to most recent vitamin D level within 1 year being deficient (<20 ng/mL); dose was decreased after last visit. Vitamin D dose was rank-ordered as follows: calcitriol > 3000+ IU D3 > 2000 IU D3 > D2 > 1-1000 IU D3/multivitamin > no vitamin D.
[d]Answer was no to most recent vitamin D level within 1 year being deficient (<20 ng/mL); dose was stable or increased after last visit. Vitamin D dose was rank-ordered as follows: calcitriol > 3000+ IU D3 > 2000 IU D3 > D2 > 1-1000 IU D3/multivitamin > no vitamin D.

© 2020 Meltzer DO et al. *JAMA Network Open.*

eTable 2. Bivariate analysis of most recent active vitamin D treatment 14 days before COVID-19 test order and COVID-19 test results, among patients with most recent vitamin D in past year<20 ng/ml

Most recent active vitamin D treatment prior to COVID-19 test order	Total patients	COVID-19 positive		COVID-19 negative	
	N	N	%	N	%
No Vitamin D	80	14	18	66	83
1-1000 IU D3/Multivitamin	28	7	25	21	75
2000 IU D3	7	<5		6	
3000+ IU D3	10	<5		8	
D2	44	7	16	37	84
Calcitriol	<5	<5		<5	
Total	172	32	19	140	81
Fisher's exact test p = 0.83					

Frequency counts of less than 5 have been replaced with the notation <5 to reduce the potential to identify members of the study sample.

© 2020 Meltzer DO et al. *JAMA Network Open.*

eReference

1. Healthcare Cost and Utilization Project (HCUP). Elixhauser Comorbidity Software for ICD-10-CM (beta version).Version 2020 v1. Accessed April 8, 2020. https://www.hcup-us.ahrq.gov/toolssoftware/comorbidityicd10/comorbidity_icd10.jsp

© 2020 Meltzer DO et al. *JAMA Network Open.*
